# Serum Calprotectin, CD26 and EGF to Establish a Panel for the Diagnosis of Lung Cancer

**DOI:** 10.1371/journal.pone.0127318

**Published:** 2015-05-18

**Authors:** Sonia Blanco-Prieto, Lorena Vázquez-Iglesias, Mar Rodríguez-Girondo, Leticia Barcia-Castro, Alberto Fernández-Villar, María Isabel Botana-Rial, Francisco Javier Rodríguez-Berrocal, María Páez de la Cadena

**Affiliations:** 1 Department of Biochemistry, Genetics and Immunology, Facultad de Biología, Universidad de Vigo, Vigo, Spain; 2 SiDOR (Statistical Inference, Decision and Operations Research) Research Group & Centro de Investigaciones Biomédicas (CINBIO), Facultad de Ciencias Económicas y Empresariales, Universidad de Vigo, Vigo, Spain; 3 Unit of Interventional Broncopleural Pathology, Pneumology Department of Complejo Hospitalario Universitario de Vigo (CHUVI), Vigo, Spain; Ospedale Pediatrico Bambino Gesu', ITALY

## Abstract

Lung cancer is the most lethal neoplasia, and an early diagnosis is the best way for improving survival. Symptomatic patients attending Pulmonary Services could be diagnosed with lung cancer earlier if high-risk individuals are promptly separated from healthy individuals and patients with benign respiratory pathologies. We searched for a convenient non-invasive serum test to define which patients should have more immediate clinical tests. Six cancer-associated molecules (HB-EGF, EGF, EGFR, sCD26, VEGF, and Calprotectin) were investigated in this study. Markers were measured in serum by specific ELISAs, in an unselected population that included 72 lung cancer patients of different histological types and 56 control subjects (healthy individuals and patients with benign pulmonary pathologies). Boosted regression and random forests analysis were conducted for the selection of the best candidate biomarkers. A remarkable discriminatory capacity was observed for EGF, sCD26, and especially for Calprotectin, these three molecules constituting a marker panel boasting a sensitivity of 83% and specificity of 87%, resulting in an associated misclassification rate of 15%. Finally, an algorithm derived by logistic regression and a nomogram allowed generating classification scores in terms of the risk of a patient of suffering lung cancer. In conclusion, we propose a non-invasive test to identify patients at high-risk for lung cancer from a non-selected population attending a Pulmonary Service. The efficacy of this three-marker panel must be tested in a larger population for lung cancer.

## Introduction

Lung cancer (LC) is the most fatal neoplasia accounting for 18% of the total cancer deaths [1]. Histological classification of lung tumors includes two major groups: small cell lung cancer (SCLC) and non-small cell lung cancer (NSCLC), which represents 75–80% of newly diagnosed lung cancers [2]. At diagnosis a substantial proportion of patients shows tumor spread and an extremely poor prognosis, with a 5-year survival rate of 16% for NSCLC [3]. Nevertheless, survival markedly varies depending on the stage, from 52% in local disease to a dramatic 4% for advanced stage [4].

The National Cancer Institute conducted the National Lung Screening Trial that demonstrated a 20% decrease in LC mortality for a high-risk population referred to screening with low-dose computerized tomography (CT) [5]. Accordingly, existing US guidelines affirm that sufficient evidence supports the implementation of LC screening with CT [6]. On the other side, uncertainty remains within Europe pending for the pooled estimation of mortality based on the ongoing screening trials [7]. The choice of which risk groups should be screened is also doubtful. Preliminary results from the largest European lung cancer screening trial, NELSON, have shown favorable stage distribution with 70.8% of individuals being diagnosed at stage I, which hopefully will result in a reduction of mortality [8].

However, a major concern of LC screening with CT is the high rate of false positive results that make this technology cost-ineffective, implying additional diagnostic procedures [9]. As a consequence, risk prediction models incorporating genetic and molecular biomarkers for early diagnosis are gaining interest to preselect patients to be submitted to low-dose CT [10, 11].

Recently, panels of serum markers to define high-risk populations for LC and specifically for NSCLC have been reported. Planque *et al*. [12] established a model with several kallikreins, while other authors have screened arrays of biomarkers with a documented implication in LC [13–15].

We analyzed levels of 6 candidate markers in serum samples collected from lung cancer patients and compared them to a control group composed of both healthy controls and individuals with benign affections of the lung. The selected molecules are cancer-associated markers covering a broad range of functionalities involved in cancer development and progression: the soluble form of Epidermal Growth Factor Receptor (sEGFR) [16]; two of the ligands that bind the EGFR: Epidermal Growth Factor (EGF) and Heparin-Binding Epidermal Growth Factor (HB-EGF); the Vascular Endothelial Growth Factor (VEGF) as one of the main executors of angiogenesis [17]; sCD26, the soluble form of CD26, a cell surface glycoprotein bearing serine protease activity and involved in immune regulation and cancer [18–20]; and an inflammation-associated molecule, Calprotectin (CAL) [21], up-regulated in several cancers, including lung [22].

The goal of our study is to obtain a novel and accurate panel of markers for discriminating patients at high risk for LC. Serum levels of the six analytes were measured, and a two-step statistical procedure was applied to derive an optimal classification algorithm. A classification score for each patient was determined, allowing an improved selection of patients for further diagnostic procedures.

## Materials and Methods

### Clinical Samples

The individuals prospectively enrolled in the present study are patients with respiratory symptoms attending the Pulmonary Service of Complejo Hospitalario Universitario de Vigo (Spain) between May 2007 and November 2010.

Eight mL of peripheral blood were collected in sterile tubes containing heparin and gelose, at the first visit. Samples were centrifuged at 3,000 rpm for 15 minutes, and serum was stored at -20°C until analysis. Patient data and serum were obtained in full compliance with the clinical-ethical practices of the Spanish Government and the Helsinki Declaration, and Galician Ethical Committee for Clinical Research approved the study. Written informed consent was provided from individuals and anonymity was warranted.

Clinical diagnostic work-up followed the recommendations of current clinical guides [23–25]. Diagnosis of LC was achieved by histological assessment of tumors following the criteria of the WHO in 1999 [26]. Staging was determined according to the 5^th^ edition of the TNM system in use at the moment the study was carried out [27]. Patients with relapse or progression of a cancer diagnosed previously and administration of chemo- or radiotherapy treatment were excluded from the study. The cancer group included 72 lung cancer cases: the median age of the patients was 71 years (range 47–88), 58 were males and 14 females, and smoking occurred in approximately 90%. NSCLC accounted for 64 cases while 8 were diagnosed as SCLC. Within the NSCLC type, adenocarcinoma was the predominant histological subtype (34 patients) and stage IV was the most frequent stage (28 cases; stages I and III 17 cases each and 2 stage II tumors).

The control cohort consisted of 56 individuals separated in two groups: one control subgroup included patients with benign pathologies of the lung such as infectious diseases (n = 31) and one patient with interstitial lung disease The other subgroup consisted of healthy controls (n = 24) and included patients who were clinically checked based on respiratory symptoms like cough, dyspnea or thoracic pain, but without specific symptomatology for LC. The median age of this control cohort was 60 years (range 24–88), composed of 33 males and 23 females, and presenting 64% of the controls smoking habits. Clinical and pathological characteristics for LC patients and individuals without LC are outlined in S1 Table.

### Measurement of Serum Biomarkers Concentration

Measurement of biomarker concentrations was conducted using commercially available enzyme-linked immunosorbent (ELISA) assays, in accordance to the respective manufacturer's suggested protocols. HB-EGF, EGF and EGFR assays were purchased from R&D Systems (Minneapolis, MN); sCD26 and VEGF from eBioscience (Ireland, UK) and the CAL assay from Hycult Biotechnology (Uden, The Netherlands). Both standard and serum samples were assayed in duplicate. Absorbance readings were collected on an EnVision Multilabel Plate Reader (Perkin Elmer).

### Statistical Methods

#### Individual Biomarker Evaluation

Continuous variables are presented as median (range), and categorical variables as frequencies (percentages). The non-parametric Mann-Whitney U and Kruskal-Wallis tests were used to assess differences on concentration of each biomarker among LC and control groups, and to perform pairwise comparisons between controls and cases subgroups. Receiver Operating Characteristic (ROC) curves and Pearson's correlation among biomarkers levels were also calculated. *P*-values<0.05 were considered statistically significant and corrected by the Holm method [28] to prevent for inflation of the type I error due to multiple testing in the subgroup analyses (simultaneously considering the subgroups defined by LC staging and control subtypes). Univariate analyses were conducted using the statistical software SPSS 15.0 (SPSS Inc., Chicago, IL) and R (Wirtschafts Universität, Wien, Austria).

#### Multivariate Panel Selection Analysis

Marker concentrations were log_10_-transformed before multivariate analysis to reduce the skewness. We used a two-step procedure, which combines variable reduction and model fit using logistic regression.

Boosted regression [29, 30] and random forests [31] were conducted for reduction of the number of markers. We used a functional gradient descent boosting approach where the fitting problem is reinterpreted as the empirical minimization of a pre-defined loss function. Minimization is achieved by repeatedly (m iterations) fitting regression trees to the negative gradients of the loss function.

Random forests rely on bootstrap aggregation. Namely, numerous regression trees are grown for each subset of biomarkers and each tree is used to predict the group membership for each observation. These are counted as “votes” for that group membership and a given observation is assigned to the group with highest number of votes.

For both methods, we considered 1,000 trees and out-of-bag (OOB) error rates were derived considering training sets with the 75% of the cases and tests sets containing the remaining 25%. For boosted regression, m acts like a tuning parameter and it is determined by minimization of the out-of-bag (OOB) error rate. Similarly, in random forests, trees are fitted in the training sets and are subsequently used for predicting the group membership of the 25% test cases. We refitted each model 1,000 times and we reported a ranking of the biomarkers according to their median relative importance [32].

We fitted all the possible logistic regression models based on the highest ranked biomarkers. Several performance indexes were calculated and compared among models: Akaike's information criterion (AIC), Bayesian information criterion (BIC) and mean squared error (MSE) as measures of lack of fit (lower values indicate better fit) and the area under the ROC curve (AUC) as a discrimination measure. Whilst AIC and BIC penalize model complexity, favoring simpler models, MSE and AUC focus on the predictive ability itself, favoring more complex models.

We used OOB predictions and we provide average values over 1,000 repetitions. All the logistic regression models included age and gender to adjust for potential confounding. Finally, we provide a nomogram graphical representation.

To evaluate the diagnostic ability of the panel indicated as best according to the aforementioned indexes (AIC, BIC, MSE and AUC), the terms of sensitivity and specificity were provided. Sensitivity refers to the percentage of patients with lung cancer that result positive with the test, in other words the capability of the test to detect the disease; the specificity is the percentage of non-cancerous individuals in which the test is negative.

All multivariate statistical analyses were carried out with the statistical software R (Wirtschafts Universität, Wien, Austria).

## Results

### Analysis of Serum Markers in Patients with Lung Cancer and in Control Subjects

Serum concentrations along with univariate statistical analyses comparing control and LC groups for each of the six biomarkers are presented in Table 1. Significantly increased serum concentrations of EGF (*p*<0.001), sEGFR (*p* = 0.037) and CAL (*p*<0.001) were found in LC patients compared to the control group, whereas sCD26 was reduced (*p*<0.001). Interestingly, only sCD26 (*p* = 0.045) and CAL levels (*p*<0.001) in benign pathologies conserved significant distinction from levels in LC, while for EGF this distinction did not reach significance (*p* = 0.100).

**Table 1:** Distribution of markers in serum of Lung Cancer patients and Controls, and efficacy in classifying Lung Cancer.

Marker	Control/Case		Median	Range	*p* ^a^	AUC (95% CI)^b^
HB-EGF (pg/mL)	**Control**		**196.50**	**32.00–4661.00**	**0.154**	**0.585 (0.435–0.755)**
		Healthy	174.50	65.00–1627.00		
		Benign	218.50	32.00–4661.00		
	**LC**		**182.50**	**24.00–1823.00**		
EGF (pg/mL)	**Control**		**340.82**	**98.01–1160.42**	**<0.001**	**0.701 (0.525–0.853)**
		Healthy	247.61	98.01–659.01		
		Benign	404.76	102.04–1160.42		
	**LC**		**577.18**	**55.97–1176.89**		
sEGFR (ng/mL)	**Control**		**34.13**	**20.82–49.57**	**0.037**	**0.619 (0.471–0.786)**
		Healthy	30.42	20.82–47.70		
		Benign	37.82	25.13–49.57		
	**LC**		**37.54**	**21.90–97.40**		
sCD26 (ng/mL)	**Control**		**473.00**	**122.00–998.00**	**<0.001**	**0.711 (0.453–0.865)**
		Healthy	504.50	331.00–816.00		
		Benign	405.50	122.00–998.00		
	**LC**		**358.50**	**136.00–1192.00**		
VEGF (pg/mL)	**Control**		**542.70**	**39.73–2631.56**	**0.055**	**0.600 (0.449–0.769)**
		Healthy	542.70	39.73–1178.40		
		Benign	541.29	124.94–2631.56		
	**LC**		**619.99**	**81.54–1856.40**		
CAL (ng/mL)	**Control**		**129.44**	**33.13–421.23**	**<0.001**	**0.781 (0.627–0.916)**
		Healthy	116.55	39.16–274.70		
		Benign	141.93	33.13–421.23		
	**LC**		**221.21**	**48.33–482.89**		

^a^ Mann-Whitney U (two-sided test).

^b^ AUC based on out-of-bag predictions (models are fitted in the training sets with the 75% of the cases and are subsequently used for predicting the group membership of the 25% test cases). Average values and percentile 95% CI over 1000 repetitions are provided.

The potential clinical usefulness of these 6 analytes as biomarkers for LC was assessed using univariate ROC curve analyses (Table 1). Remarkable discriminatory capacity was encountered for EGF and sCD26, with an Area Under the Curve (AUC) of 0.701 and 0.711, respectively; CAL exhibited the most promising profile with an AUC of 0.781.

Correlation among markers was also assessed revealing that several molecules were slightly correlated with each other (data not shown). Only for sCD26 and CAL with VEGF, the correlation was >0.3.

### Analysis of Serum Markers according to the Tumor Classification

Levels of the markers were also analyzed in NSCLC patients (89% of LC cases) according to the tumor spread (Table 2). Among significant discriminant markers, EGF and CAL levels were already statistically distinguishable from healthy and benign controls at early stages I-II (*p*<0.001 and *p* = 0.002, respectively). These markers also displayed significant differences between NSCLC stages III-IV patients and controls (*p* = 0.012 for EGF and *p*<0.001 for CAL), in spite of EGF displaying notable inferior levels in advanced stages as compared to earlier stages. On the other side, no statistically significant differences were found between sCD26 levels at early stages and those in controls (*p* = 0.116), but they effectively differed in disseminated stages in relation to controls (*p*<0.001).

**Table 2:** Distribution of markers in early and advanced NSCLC versus Controls.

Marker	Control/ Case		Median	Range	*p* ^a^
HB-EGF (pg/mL)	**Control**		**196.50**	**32.00–4661.00**	
	**NSCLC**				
		NSCLC I+II	159.00	56.00–435.00	0.630
		NSCLC III+IV	190.00	24.00–1823.00	0.698
EGF (pg/mL)	**Control**		**340.82**	**98.01–1160.42**	
	**NSCLC**				
		NSCLC I+II	792.82	388.23–1176.89	<0.001
		NSCLC III+IV	477.74	144.23–1158.32	0.012
sEGFR (ng/mL)	**Control**		**34.13**	**20.82–49.57**	
	**NSCLC**				
		NSCLC I+II	38.28	29.17–46.44	0.375
		NSCLC III+IV	37.14	21.90–46.28	0.375
sCD26 (ng/mL)	**Control**		**473.00**	**122.00–998.00**	
	**NSCLC**				
		NSCLC I+II	396.00	206.00–640.00	0.116
		NSCLC III+IV	356.00	136.00–945.00	<0.001
VEGF (pg/mL)	**Control**		**542.70**	**39.73–2631.56**	
	**NSCLC**				
		NSCLC I+II	469.86	227.00–1353.08	0.990
		NSCLC III+IV	663.85	81.54–1856.40	0.028
CAL (ng/mL)	**Control**		**129.44**	**33.13–421.23**	
	**NSCLC**				
		NSCLC I+II	196.16	120.73–426.99	0.002
		NSCLC III+IV	247.06	107.71–482.89	<0.001

^a^ Mann-Whitney U (two-sided test) for comparison among the control group and early and advanced NSCLC stages. P-values were corrected by the Holm method to prevent for inflation of the type I error due to multiple testing (corrections based on multiplicity given by controls subtypes and LC staging).

For the remaining markers, stratification by tumor extension did not result in differences with the control group, with the sole exception of VEGF. When stratified by tumor stage, sEGFR lost its discrimination from controls, both at early stages I-II, and at advanced stages III and IV (*p* = 0.375 for both comparisons). Conversely, for VEGF the pronounced increase in its levels with disease progression led to significantly higher levels at progressive stages III-IV than those in the control group (*p* = 0.028).

S2 Table provides a deeper analysis of marker levels in individual NSCLC stages. For markers that proved significant discriminators between control subjects and LC patients overall, sCD26 and CAL showed a quite homogeneous trend to worsening of their levels with NSCLC progression, which accounted for the differentiation of late NSCLC regarding controls in the case of sCD26. Remarkably, the trend for EGF levels was the opposite, with levels in advanced stages approaching those exhibited by control subjects.

Although a detailed analysis according to the tumor extension was not possible in the SCLC group due to the reduced sample size, there was a notable difference between levels at limited and extended disease for HB-EGF (111.00 *versus* 236.00 pg/mL), EGF (78.37 *versus* 447.90 pg/mL) and CAL (97.32 *versus* 313.00 ng/mL). Similar levels were encountered for limited and extended stages in the case of sEGFR (41.96 *versus* 35.22 ng/mL), sCD26 (339.00 *versus* 294.00 ng/mL) and VEGF (530.82 *versus* 493.76 pg/mL).

**Fig 1:**
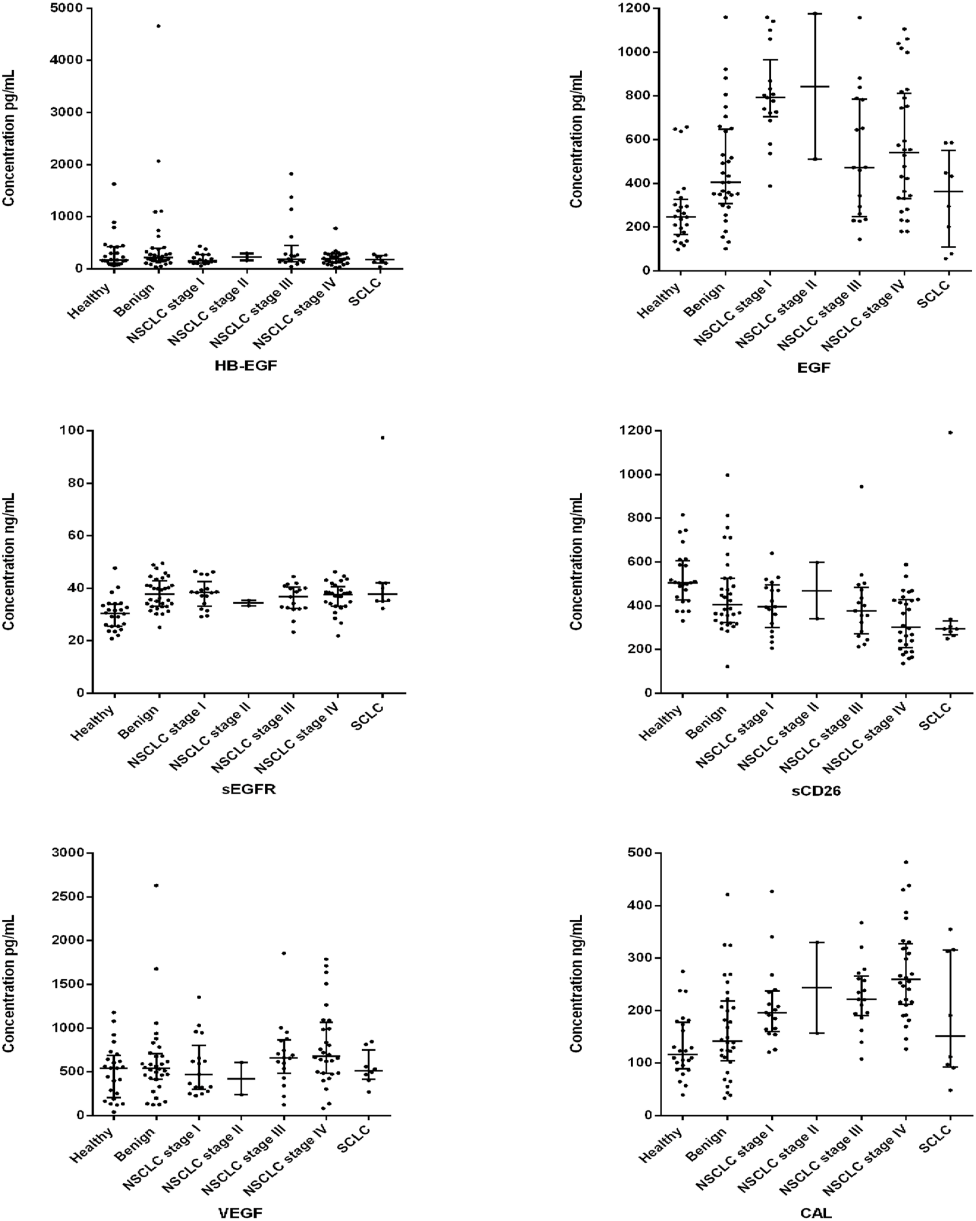
Box-plots of the 6 biomarkers. Box-plots of the levels of the six biomarkers candidates in the sera subgroups of controls and lung cancer patients. Horizontal lines represent median values.

A graphical display of individual biomarker profiles considering all the subgroups of patients is presented as box-plots in Fig 1.

### Comparison of Biomarkers with Demographic Parameters

Levels of HB-EGF, EGFR, VEGF and CAL did not show any relationship with demographic parameters. EGF (*p* = 0.004) and sCD26 (*p* = 0.014) levels varied significantly by gender; furthermore, sCD26 levels diminished progressively with age (*p* = 0.001). Solely EGF correlated to smoking status, with levels notably superior in smokers (*p* = 0.011) (Table 3).

**Table 3:** Relationship between marker levels and demographic parameters. .

Parameter		Marker^a^					
		HB-EGF (pg/mL)	EGF (pg/mL)	sEGFR (ng/mL)	sCD26 (ng/mL)	VEGF (pg/mL)	CAL (ng/mL)
Gender							
	Male (n = 91)	191.00(24.00–1823.00)	528.51(55.97–1176.89)	35.71(20.82–97.40)	375.00(136.00–1192.00)	594.40(39.73–1856.40)	191.48(39.16–438.32)
	Female (n = 37)	188.00(44.00–4661.00)	334.55(116.45–868.94)	34.20(22.05–45.77)	440.00(122.00–945.00)	549.87(81.54–2631.54)	185.67(33.13–482.89)
	*p* ^b^	0.902	0.004	0.526	0.014	0.106	0.271
Age							
	<65 yr (n = 67)	191.00(24.00–2067.00)	473.71(78.37–1176.89)	38.32(20.82–49.57)	463.00(159.00–998.00)	565.82(39.73–1711.25)	185.67(38.67–438.32)
	>65 yr (n = 60)	189.50(32.00–4661.00)	426.97(55.97–1142.11)	34.55(23.32–97.40)	348.50(122.00–1192.00)	587.58(81.54–2631.56)	192.81(33.13–482.89)
	*p* ^b^	0.714	0.443	0.086	0.001	0.372	0.487
Smoking							
	Yes (n = 85)	191.00(24.00–1823.00)	579.85(55.97–1176.89)	36.77(21.90–97.40)	377.00(136.00–1192.00)	594.40(39.73–1856.40)	207.88(38.67–438.32)
	No (n = 19)	188.00(32.00–4661.00)	344.68(98.01–1160.42)	34.20(20.82–47.67)	425.00(122.00–998.00)	616.56(81.54–2631.56)	181.44(33.13–482.89)
	*p* ^b^	0.680	0.011	0.635	0.207	0.665	0.498

^a^ Median and range values provided.

^b^ Mann-Whitney U (two-sided test)

### Selection of a Multi-marker Panel for Detection of Lung Cancer

Using the boosting and random forests methods, each marker was ranked by importance for predicting LC (Table 4). Both methods allow for a ranking of markers that mostly contribute to distinction between cancer and control subjects. According to the boosting method, CAL showed superior relative importance, followed by sCD26 and EGF with a similar relative importance; random forest mainly coincides in their ranking. In light of these results we chose CAL, sCD26 and EGF as the relevant biomarkers for detecting LC.

**Table 4:** Ranking of each marker according to its importance for predicting Lung Cancer.

Marker	Boosted Regression			Random Forests		
	Rank	Median Importance^a^	Range	Rank	Median Importance^a^	Range
CAL	1	69	56–82	1	10	9–10
sCD26	2	16	6–31	2	7	6–8
EGF	3	13	5–24	3	6	6–7
sEGFR	4	2	0–7	4	5	4–5
VEGF	5	0	0–4	6	4	4–4
HB-EGF	6	0	0–1	5	5	4–5

^a^ Importance measures report method-specific estimates of the individual contribution to prediction of each evaluated biomarker. Medians values and range over 1000 runs are provided.

Next, we constructed all the possible logistic regression models based on combinations of these three markers and gender and age as potential confounding variables (Table 5). Several performance indexes were applied to investigate which model behaves best. While BIC is more conservative and identifies the model with CAL alone as the optimal one (lowest BIC value), AIC identifies the model with three markers as the optimal one (it displays the lowest AIC value), in agreement with the highest AUC and lowest prediction MSE.

**Table 5:** Performance of each model possibility.

Markers included in the model	Performance Indexes			
	AIC	BIC	MSE^a^	AUC^a^
CAL	135.39	146.77	0.176 (0.115–0.252)	0.816 (0.671–0.949)
CD26	155.27	166.65	0.214 (0.158–0.286)	0.732 (0.567–0.886)
EGF	150.25	161.63	0.205 (0.152–0.277)	0.759 (0.606–0.893)
CAL+ sCD26	133.43	147.65	0.173 (0.108–0.249)	0.823 (0.680–0.958)
CAL+ EGF	133.77	147.99	0.173 (0.110–0.252)	0.824 (0.677–0.954)
CD26+EGF	146.77	160.99	0.196 (0.138–0.274)	0.778 (0.626–0.917)
CAL+ sCD26+EGF	132.64	149.70	0.169 (0.103–0.247)	0.828 (0.683–0.962)

^a^ Area under the ROC curve (AUC) and mean squared error (MSE) based on out-of-bag (OOB) predictions (models are fitted in the training sets with the 75% of the cases and are subsequently used for predicting the group membership of the 25% test cases). Mean AUC and MSE and 95% bootstrap confidence intervals are provided.

### Classification Algorithm

As derived from performance indexes, a valuable marker panel that might be useful for LC diagnosis was established, constituted by CAL, sCD26 and EGF. The associated logistic regression model generated an algorithm to estimate a classification score (*p*) for each patient, given by the estimated probability of presenting LC, as a function of the selected biomarkers (CAL, sCD26 and EGF), defined as follows:

$$p=\frac{1}{1+{\text{e}}^{-\{\alpha_{0}+\alpha_{1}{\text{I}}_{\left\{\text{woman}\right\}}\;+\alpha_{2}\text{age}+\sum_{\text{i}=1}^{\text{q}}\beta \text{iXi}\}}}$$

Where X_1_,…, X_i_ are the logarithmically transformed (base 10) concentrations of markers, α_0_ is the specific constant of the model and α and β are the coefficients for demographic variables (age and gender) and each of the selected biomarkers, respectively.

### Diagnostic Performance of the Selected Panel of Markers

With the aim of estimating the diagnostic parameters of the selected three-marker classification algorithm in our patient population, the values for the classification score *p* were generated for the individuals evaluated in our study. Different cut-off points for *p* with the associated sensitivities and specificities were proposed (Table 6). The optimal threshold for discrimination of cancerous from non-cancerous patients was 0.559, meaning that a patient with a score higher is considered to have lung cancer and one patient with a score inferior to 0.559 is considered not to have cancer, this cut-off carries a sensitivity of 83% and a specificity of 87%.

**Table 6:** Diagnostic performance of the panel composed off CAL+sCD26+EGF+gender+age.

Cut-off point	Sensitivity (%)	Specificity (%)
**0.559** ^a^	**83**	**87**
0.410	90	62
0.107	95	18
0.076	98	17
0.772	57	90
0.839	39	95
0.927	13	98

^a^ Dichotomization of the classification score at this cut-off resulted in the best terms of sensitivity and specificity.

### Impact of Demographic and Clinical Variables on Misclassification by the Prediction Model

The overall misclassification rate for the three-marker panel, for a fixed cut-off of 0.559, was 15%. An examination of the individual groups was then performed to assess the role of demographic and clinical variables on incorrect cases classification. The panel yielded a higher misclassification rate of non-smokers (26%) than smokers (16%), due (at least in part) the confounding effect of smoking on EGF values. Regarding staging, misclassifications in early stages I-II were highly equivalent to those presented in advanced stages III-IV, 11 and 13%, respectively. SCLC cases were misclassified to a greater degree, 50% (4/8 cases), than NSCLC cases (12%). When looking solely at the cohort composed of healthy controls, only one subject was misclassified out of the 24 tested (4%), whilst 6 of 32 patients with benign lung affections were erroneously classified (19%).

### Prediction Nomogram

**Fig 2:**
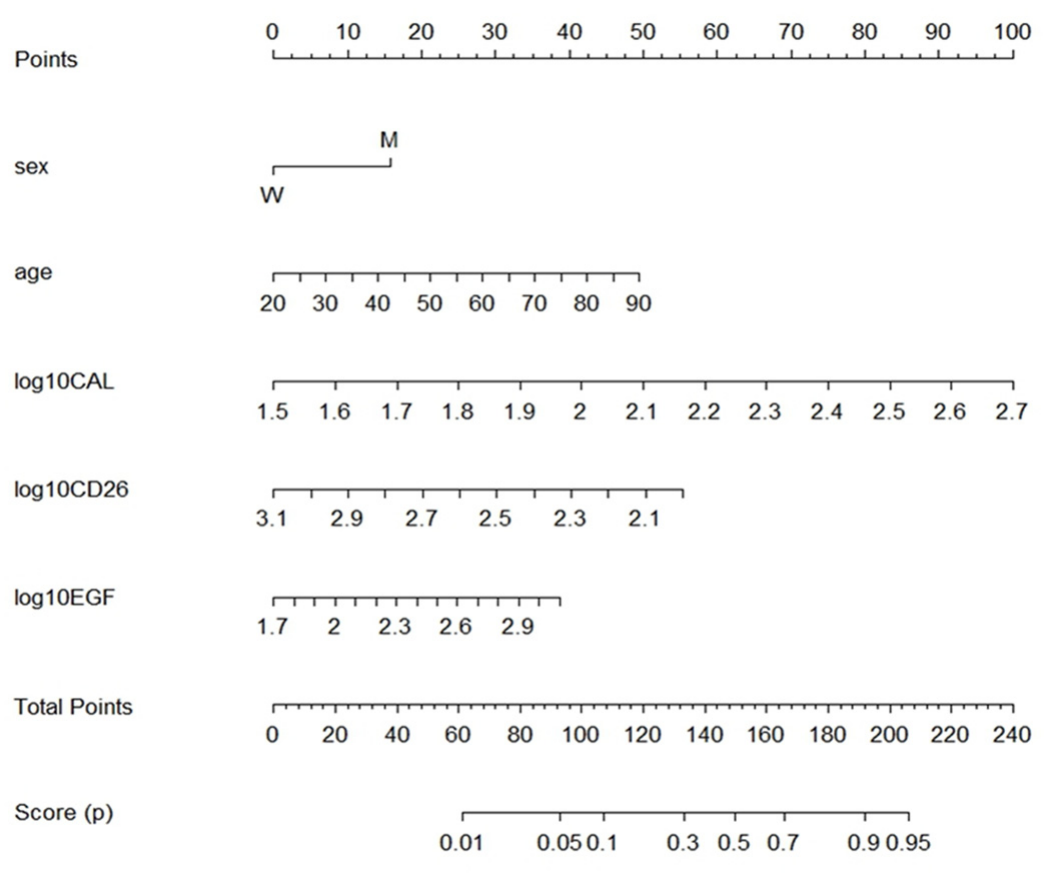
Nomogram for prediction of the classification score *p* for lung cancer. Multivariable logistic regression model-based nomogram to define lung cancer score *p* based on Calprotectin, sCD26 and EGF concentration (log transformed), gender and age.

The nomogram is a simple way to interpret a multivariate marker panel and to visualize the different patient profiles that yield to different values of the associated classification score *p*. Fig 2 shows a nomogram for the panel including CAL, sCD26 and EGF levels (log transformed). To obtain the score of a given patient, the corresponding values for each marker are located at the corresponding axis and vertically related to the “Points” axis that attributes points to the variable depending on its levels. Note that CAL is the marker associated with the highest number of points, which means that it is the variable with the highest importance to calculate the score *p*. Once calculated the contribution in points of each variable, they are summed, and the axis of total points can then be directly related to the classification score *p* axis below.

## Discussion

Our goal was to identify candidate serum markers to constitute a panel and propose a classification model to be used in the habitual symptomatic non-selected population visiting the Pulmonary Services for the selection of those patients amenable to be submitted to diagnostic procedures. Initially we included 6 molecules: HB-EGF, EGF, sEGFR, VEGF, sCD26 and CAL to investigate their potential as diagnostic markers in LC.

sEGFR, comprising only the extracellular domain of EGFR, is found in blood [33, 34] and can potentially reflect changes in tumor growth. Two of the ligands that bind to EGFR were also selected for this study: EGF and HB-EGF. Both serum EGFR and EGF demonstrated good diagnostic capacity in LC patients in a previous study [35] whilst the role of HB-EGF in LC has only been assessed in terms of response to EGFR treatment [36]. VEGF as a mediator of angiogenesis [17] has been extensively evaluated as a prognostic factor in LC [37, 38]. sCD26, the soluble form of the transmembrane protease dipeptidyl peptidase IV (DPPIV) [18] is reported at low levels in solid malignancies [20] and it is suggested as a noteworthy sensitive assay for the early diagnosis and screening of CRC [39]. Belonging to the S100 family of calcium binding proteins, CAL is an immunogenic protein abundantly expressed and released by phagocytes, exerting antimicrobial and pro-inflammatory properties [21]. A function in cancer development has been suggested due to its up-regulation in various tumors such as lung [22].

From our results we can conclude that EGF, sCD26 and CAL showed the highest discriminative capacity for lung malignancy. EGF demonstrated a relatively good performance in LC diagnosis with levels significantly increased in comparison to the control group. In a previous study (Lemos *et al*. [35]), we described statistically lower levels of EGF in serum of NSCLC patients than in controls. These apparently contradictory results are due to differences in the control and patients cohorts. Here we included benign pathologies, and healthy individuals were symptomatic ones, while in the previous work these were healthy donors. Moreover, patient´s group in the former study included mainly advanced NSCLC patients. On the other hand, the dependence of EGF levels on gender and smoking status was not reported in that study.

The results regarding sCD26 in LC diagnosis were also promising. The role of CD26 in carcinogenesis is not unequivocal. On the one side, pro-oncogenic activities mediating lung metastasis are reported [40]. On the other side, a suppressor role on lung cancer was demonstrated [41], as re-expression of CD26 reversed the malignant phenotype of non-small cell lung cancer cells. In our study, sCD26 experienced a significant reduction on its level in cancer patients compared to control individuals. Such a reduction has already been observed [42] and, likewise, an association with older age and lower sCD26 levels was encountered; also, a significant decrease on DPPIV activity with age in a population of healthy subjects had earlier been described [43]. De Chiara *et al*. [44] measured serum sCD26 in a large cohort and reported overall mean levels of 522 ng/mL (range 118–3062), in accordance to levels in our control cohort. Similarly, they observed slightly higher concentrations in women and a modest decrease in levels along decades.

CAL refers to the heterodimer formed by S100A8 and S100A9 proteins, and it is a mediator of diverse processes within chronic inflammation being high levels characteristic for inflammatory conditions. Differential expression of CAL has been shown to represent a key step contributing to cancer development and progression in malignancies of the bladder, skin, breast, gastric, colorectal, lung, pancreas, prostate and squamous esophageal carcinomas [21, 22].

In a previous work a high accuracy of CAL for predicting malignancy in patients with exudative pleural effusion has been demonstrated [45]. CAL levels in LC causing effusions (255.4 ng/mL) were similar to that reported here in serum. In contrast, much higher mean levels in benign pleural effusions (2,627.1 ng/mL) were found in comparison with mean levels in sera (141.93 ng/mL), evidencing the different nature of the fluids analyzed. A stronger tissue expression of S100A8 and S100A9 in lung malignancy compared with benign peripheral and adjacent lung tissues was reported [46], in accordance with our data.

One of the major requirements of a tumor marker is its capacity to detect the disease in early stages. EGF and CAL levels are already altered in stages I-II of NSCLC in comparison to controls, while sCD26 presented an accentuated decrease, although not significant. Similar analysis in SCLC patients could not be performed due to the low number of individuals; nevertheless, patients with limited disease exhibited EGF and CAL levels inferior to the ones in the control group that could indicate some degree of difficulty in classifying these patients. Overall, these results suggest the utility of the panel in screening campaigns. This fact evidences the major limitation of our study, the relatively small size of our cohort that precludes comparisons like the aforementioned, warranting further large-scale studies to validate these findings. The same applies for the generation of the classification model, although in this case the statistical procedures at both steps were repeated 1000 times and resulting parameters averaged, which avoids overestimation and ensures reproducibility of the model in future populations.

To reach a clinically meaningful classification algorithm for LC based on most discriminative markers a two-step statistical strategy was performed. Boosting and random forest methods allow for ranking of biomarkers according to relative variable importance indices based on the median relative influence across all the generated trees [32]. Both, boosting and random forest methods, confirmed the superiority of CAL, sCD26 and EGF. In a second step, the panel of biomarkers was used to fit logistic regression models to derive a (optimal) classification rule for assigning a patient's diagnosis. According to performance indexes applied, the panel including CAL, sCD26 and EGF was established as the most informative. The model included gender and age to correct for potential confounding, especially in the case of sCD26, whose levels are influenced by age. Since smoking data is missing for some individuals this variable could not be considered in the analysis.

It should be noted the strength of CAL both in univariate regression models, where the preferred is the one including CAL alone, and considering the combination of markers, reinforcing CAL as the most important biomarker for LC diagnosis.

Applicability of the corresponding algorithm allows obtaining a score by introducing in the model the patient´s own marker concentrations. Whether the resulting *p* score is higher than the fixed cut-off, the individual will be classified as having LC with a sensitivity and specificity relative to this cut-off. In addition, we provide a nomogram representation of the optimal multivariate model for better interpretation of the scoring system, allowing checking the possible combination of values of the biomarkers that are associated with a specific value of the score *p*.

The three-marker panel showed an overall misclassification rate of 15%. Within the classification errors, SCLC was the histology more frequently misclassified, but the number of patients is too small to extract a definite conclusion. Furthermore, among NSCLC histology groups, no important variations in misclassifications were noticed, as expected from the absence of significant differences in marker concentrations among histology classification, with the solely exception of EGF whose levels in SCLC where significantly lower than in the adenocarcinoma subtype. In the control group, the benign diseases were the most challenging to classify. Nevertheless, the correct classification of a high number of benign lung affections (81%) and of early stage LC patients (89% of stage I-II patients), increases the value of the panel.

A number of multianalyte panels have been reported to distinguish LC patients from individuals without cancer. However, differences in methodological and design aspects restrict the comparisons of efficacy among them. Blood processing protocols and, specifically, criteria for patient selection with a control group not representative enough or considering healthy blood donors are the main discrepancies. This point is precisely one of the strengths of our work: both healthy subjects and patients with common non-neoplastic affections of the lung (such as respiratory infections) commonly attending the Pulmonary Services were included in the control group. Additionally, we included besides the most frequent lung cancer type, NSCLC, small cell lung cancer (SCLC) patients.

Some of the studies with promising results for NSCLC diagnosis are hereafter discussed. Planque *et al*. [12] established a model with several kallikrein proteins that presented an AUC of 0.82. Lee *et al*. [14] investigated 30 markers in NSCLC and a healthy control cohort, by means of a multiplex platform. Multivariate classification algorithms applied rendered a 5-marker panel as the most accurate, composed of alpha-1-antitrypsin, CYFRA, Insulin Growth Factor 1, RANTES and alpha fetoprotein that in a validation cohort achieved in the logistic regression model a sensitivity of 85.1% and specificity of 95.9%. It is important to mention that a population of benign pathologies was not included in this study, unlike our work. The work of Farlow *et al*. [13] incorporated non-neoplastic lung diseases and inflammatory conditions for comparison against NSCLC and defined a six-marker panel using random forest and CART algorithms, comprised by TNF-α, CYFRA, Interleukin 1ra, Matrix Metalloproteinase-2, Monocyte chemotactic protein 1 and sE-selectin. In the classification tree a sensitivity of 99% and specificity of 95% were obtained, but in a validation series accuracy diminished with 75 of 88 patients well classified implying a 15% misclassification rate, identical to our results. In line with our data, these authors concluded that inflammatory conditions were the most difficult to assign.

The difficulty of discriminating NSCLC from subjects with non-neoplastic respiratory diseases is also reflected on the work of Izbicka *et al*. [15], who determined the plasma levels of 57 proteins to distinguish NSCLC from asthma patients and healthy controls. They found relatively small quantitative differences in markers expression when comparing NSCLC with asthma patients, and propose a four or five-marker panel for diagnosis excluding the asthma category, with sensitivity and specificity in the four best subset of markers of 93 and 87% (composed of EGF, sCD40 ligand, Interleukin 8 and Matrix Metalloproteinase-8). Similarly to us, EGF behaves as one of the strongest predictors.

Also, panels have been indicated as an aid to interpret CT scans, such as the study of Bigbee *et al*. [47], which proposed a 10-marker panel using a rule learning approach that discriminates NSCLC from controls with a sensitivity of 73% and specificity of 93%. Prolactin, Transthyretin, Thrombospondin, E-selectin, C-C motif chemokine 5 (CCL5, RANTES), Macrophage Migration Inhibitory factor (MIF), serpine 1 (PAI-1), ErbB-2, CYFRA and Serum Amyloid A protein (SAA) constituted this complex panel. VEGF, EGFR and EGF were among the initial candidates but the model did not select them.

Although a considerable number of investigations have been carried out with the aim of identifying biomarker panels for the early detection of NSCLC, only a few have been translated into clinical or commercial setting. The EarlyCTD-Lung is a blood test that measures autoantibodies to lung cancer-associated antigens [48]. It has been clinically tested for LC, demonstrating in a high-risk population of 1,600 patients 41% sensitivity and 87% specificity. The PAULA´s test (Protein Assays Using Lung cancer Analytes), offered by Genesys Biolabs [49], determines the risk of LC in patients at high-risk as an aid in the determination of appropriate diagnostic follow-up. The panel comprises three tumor antigens (CEA, CA125 and CYFRA) and one autoantibody (NY-ESO1), and a sensitivity of 74% and specificity of 80% have been reported. The INDI Company has recently published a 13-protein classifier [50], based on Multiple Reaction Monitoring and mass spectrometry (MRM-MS), and a cancer score intended to differentiate benign nodules and rescue them from unnecessary procedures. Reported performance for this classifier is a negative predictive value of 95% and a specificity of 66%.

In conclusion, among the 6 cancer-related proteins evaluated for its diagnostic ability in LC, we have established a detection algorithm based on a three-marker panel achieving similar accuracies to those reported in other studies. sCD26 and CAL showed remarkably diagnostic value, not previously reported, whereas EGF and CAL demonstrated to be valuable in early diagnosis of LC. This approach could serve as a promising low-cost and minimally invasive test to select patients at high-risk for LC as a complement to CT or to indicate further diagnostic procedures, with individuals presenting scores over the cut-off being submitted to more immediate tests (biopsy, surveillance…). Relevance of this algorithm needs to be validated in a larger population, preferably in a lung cancer screening population, while further addition of complementary biomarkers could optimize the algorithm in terms of sensitivity and specificity.

## Supporting Information

S1 Table Patient Demographics and Clinical Profiles. (DOCX)

S2 Table Distribution of markers in NSCLC stages and Controls. (DOCX)
