# Supplementary material for: Serum Calprotectin, CD26 and EGF to Establish a Panel for the Diagnosis of Lung Cancer
Source: PLoS One. 2015 May 18;10(5):e0127318. doi: 10.1371/journal.pone.0127318 (PMC4436352; doi:10.1371/journal.pone.0127318)
Supplement: S1 Table — (DOCX) [file pone.0127318.s001.docx]

**S1 Table. Patient Demographics and Clinical Profiles**

|  |  |  | **Cases (n=72)** | **Controls (n=56)** |
| --- | --- | --- | --- | --- |
| Gender | Male |  | 58 | 33 |
|  | Female |  | 14 | 23 |
| Age^a^ |  |  |  |  |
|  | Median |  | 71 | 60 |
|  | Range |  | 47-88 | 24-88 |
| Smoking Status^b^ |  |  |  |  |
|  | Yes |  | 64 | 21 |
|  | No |  | 7 | 12 |
| Diagnosis |  |  |  |  |
|  | NSCLS |  |  |  |
|  |  | ADC | 34 |  |
|  |  | SCC | 17 |  |
|  |  | LCC | 11 |  |
|  |  | ND | 1 |  |
|  |  | BAC | 1 |  |
|  | SCLC |  | 8 |  |
|  | Healthy |  |  | 24 |
|  | RI |  |  | 31 |
|  | EPID |  |  | 1 |
| Stage |  |  |  |  |
|  | NSCLC |  |  |  |
|  |  | I | 17 |  |
|  |  | II | 2 |  |
|  |  | III | 17 |  |
|  |  | IV | 28 |  |
|  | SCLC |  |  |  |
|  |  | Limited | 3 |  |
|  |  | Extended | 5 |  |

Abbreviations: NSCLC= non-small cell lung cancer, ADC= adenocarcinoma, SCC= squamous cell carcinoma, LCC= large cell carcinoma, SCLC= small-cell lung cancer, ND= not differentiated, BAC= bronco-alveolar carcinoma, RI= respiratory infection, EPID= diffuse interstitial lung disease

^a^ Statistically different between the case and control group, *P*=0.001(Mann-Whitney U test)

^b^ Unknown data on smoking status for cases (n=1) and for controls (n=23)
