# Supplementary material for: Serum Calprotectin, CD26 and EGF to Establish a Panel for the Diagnosis of Lung Cancer
Source: PLoS One. 2015 May 18;10(5):e0127318. doi: 10.1371/journal.pone.0127318 (PMC4436352; doi:10.1371/journal.pone.0127318)
Supplement: S2 Table — (DOCX) [file pone.0127318.s002.docx]

**S2 Table. Distribution of markers in NSCLC stages and Controls**

| Marker | Control/Case |  | Median | Range |
| --- | --- | --- | --- | --- |
| HB-EGF (pg/mL) | **Control** |  | **196.50** | **32.00-4661.00** |
|  | **NSCLC** |  |  |  |
|  |  | NSCLC I | 156.00 | 56.00-435.00 |
|  |  | NSCLC II | 228.00 | 159.00-297.00 |
|  |  | NSCLC III | 190.00 | 44.00-1823.00 |
|  |  | NSCLC IV | 194.00 | 24.00-776.00 |
| EGF (pg/mL) | **Control** |  | **340.82** | **98.01-1160.42** |
|  | **NSCLC** |  |  |  |
|  |  | NSCLC I | 792.82 | 388.23-1159.73 |
|  |  | NSCLC II | 844.19 | 511.49-1176.89 |
|  |  | NSCLC III | 472.90 | 144.23-1158.32 |
|  |  | NSCLC IV | 541.27 | 180.70-1106.52 |
| sEGFR (ng/mL) | **Control** |  | **34.13** | **20.82-49.57** |
|  | **NSCLC** |  |  |  |
|  |  | NSCLC I | 38.38 | 29.17-46.44 |
|  |  | NSCLC II | 34.35 | 33.34-35.36 |
|  |  | NSCLC III | 36.77 | 23.32-44.47 |
|  |  | NSCLC IV | 37.54 | 21.90-46.28 |
| sCD26 (ng/mL) | **Control** |  | **473.00** | **122.00-998.00** |
|  | **NSCLC** |  |  |  |
|  |  | NSCLC I | 396.00 | 206.00-640.00 |
|  |  | NSCLC II | 469.50 | 341.00-598.00 |
|  |  | NSCLC III | 377.00 | 213.00-945.00 |
|  |  | NSCLC IV | 303.00 | 136.00-588.00 |
| **VEGF (pg/mL)** | **Control** |  | **542.70** | **39.73-2631.56** |
|  | **NSCLC** |  |  |  |
|  |  | NSCLC I | 469.86 | 227.00-1353.08 |
|  |  | NSCLC II | 423.65 | 239.65-607.65 |
|  |  | NSCLC III | 661.29 | 122.53-1856.40 |
|  |  | NSCLC IV | 678.98 | 81.54-1787.40 |
| CAL (ng/mL) | **Control** |  | **129.44** | **33.13-421.23** |
|  | **NSCLC** |  |  |  |
|  |  | NSCLC I | 196.16 | 120.73-426.99 |
|  |  | NSCLC II | 243.49 | 156.84-330.15 |
|  |  | NSCLC III | 221.36 | 107.71-367.73 |
|  |  | NSCLC IV | 259.27 | 126.50-482.89 |
